# Wild Birds’ Genetic Resources Bank: Feather Follicle Cell Culture as a Possible Source of Stem Cells

**DOI:** 10.3390/mps8010017

**Published:** 2025-02-08

**Authors:** Yasmin Godoi dos Reis, Maria Eduarda Pralon Guerra, Meline de Paula Coutinho, Sarah Ingrid Pinto Santos, Bruna Dias Mota, Lauriene Luiza de Souza Munhoz, Diogo Pascoal Rossetti, Daniele dos Santos Martins

**Affiliations:** 1 Laboratory of Immunohistochemistry and Experimental Physiology, Department of Veterinary Medicine, School of Animal Science and Food Engineering, University of São Paulo, Pirassununga 13635-900, SP, Brazil; yasmingodoireis@gmail.com (Y.G.d.R.); dudapralongr@gmail.com (M.E.P.G.); brunadiasmota@usp.br (B.D.M.); lauremunhoz@usp.br (L.L.d.S.M.); 2 Laboratory of Stem Cells and Gene Therapy, Department of Veterinary Medicine, School of Animal Science and Food Engineering, University of São Paulo, Pirassununga 13635-900, SP, Brazil; melinecoutinho@usp.br (M.d.P.C.); sarahingridpsantos@gmail.com (S.I.P.S.); 3 Zoo das Aves, Poços de Caldas 37704-397, MG, Brazil; diogo.rossetti@zoodasaves.com.br

**Keywords:** feather follicle cell culture, wild birds, biobanks

## Abstract

Follicular cells represent a valuable resource for genetic research, biotechnology and cryopreservation in biobanks, particularly for the conservation of endangered species. They offer a more practical alternative to gametes, embryos and fibroblasts. Collection of these cells can be achieved through feather plucking. Feather samples were opened with a scalpel and the feather pulp was washed with PBS, cut into cubes and digested in collagenase type IV. Cultivation was carried out in DMEM culture medium with 15% fetal bovine serum, 1% penicillin/streptomycin and 0.5% amphotericin, under incubation conditions of 39.5 °C and 5% CO_2_. Passages were carried out with 5% EDTA for 5 min. The culture was successful, with great cell proliferation, adherence to plastic and aggregation into cell colonies. This method was effective in obtaining feather follicle cells from wild birds, especially when collected up to 6 h after their death, and can serve as a base protocol for research with feather follicle cells aiming to create biobanks.

## 1. Introduction

The deterioration of the natural environment, coupled with a multitude of additional factors, has precipitated the decline and fragmentation of wildlife populations. As a strategy to overcome this, samples from various wild species have been systematically collected and cryopreserved. This has been performed to conserve genetic diversity and improve the management of captive (ex situ) and wild (in situ) animals [1]. Among the threatened populations are wild birds, which are subject to the loss of genetic diversification that, in extreme situations, can lead to the extinction of more vulnerable species. However, advances in cell culture technology have made it possible to obtain bird cells with the potential for genetic reprogramming and the generation of germ cells [2].

Adult stem cells can be derived from somatic tissues, including skin, bone marrow, blood, adipose tissue, synovial membrane, umbilical cord and amniotic fluid. These cells are multipotent; therefore, they could be differentiated into various functional cell types. Additionally, they possess the ability to proliferate and self-renew, rendering them invaluable for genetic research, biotechnologies and innovative therapies [3].

Many stem cell harvesting techniques involve invasive protocols that require a hospital environment and anesthesia to obtain the source tissue. Some sources even require the animal to be euthanized. The scientific literature lists a variety of sources for the isolation of avian stem cells, including lung [4], liver [5], bone marrow [6], umbilical cord, Wharton’s jelly [7] and amnion [8]. This highlights the invasive nature of the procedure. A fibroblast-like cell culture was first obtained from feather pulp by Sasaki, Ikeuchi and Makino [9]. This is a less invasive source of cells and therefore more interesting for species conservation. Later, Xi [10] and Cardoso [2] used samples of chicken feather pulp and succeeded in obtaining a culture of epidermal stem cells. Xu’s [11] (2011) subsequent investigation delved deeper into the characteristics of the cells obtained from the feather follicle, showing that they presented stem cell properties, including self-renewal and multi-potential. This led to the noteworthy conclusion that these cells could be considered as an epidermal stem cell source in adult birds. Kim [12] characterized the fibroblast-like cells from the feather follicle with mesenchymal stem cell (MSC) markers such as CD29, CD44, CD90 and CD105, analyzed their proliferation capacity and differentiation potential and concluded that the feather follicular cells (FFCs) present the same characteristics as other MSCs. All these studies isolated cells from the feather pulp, inside the growing calamus. This method is less invasive but still very efficient. Some authors consider it to be the most accessible source of avian somatic stem cells [12].

The feather follicle refers to the invaginated epidermis that surrounds a sheath with the feather filaments and pulp inside. Pulp is defined as a central axial artery containing abundant proliferating mesenchymal cells, located at the base of the follicle. Structurally, the pulp is composed of a dermal papilla and an area above this, still in the pulp region, but in an area called the collar, where adjacent epithelial stem cells are located [13]. This property, akin to that of hair, renders feathers well-suited for undergoing molting cycles and regenerating themselves. This attribute lends them a compelling application in various studies as a model for stem cell research [14]. Therefore, it is important to note that the use of somatic cell cultures has provided a new alternative for biobanks, as they can be used to develop induced stem cells (iPSCs), as well as somatic cell nucleus transfer (SCNT) and the production of chimeric animals through cell transplantation [15,16].

This alternative is particularly salient in light of the challenges associated with the limited acquisition and preservation of gametes and embryos from birds. Freezing is unpractical due to the substantial amount of yolk present in the oocyte and zygote. Additionally, sperm exhibit unique physiological characteristics in their cell membranes that result in post-cryopreservation modifications. This underscores the necessity to establish stem cells as alternative sources of germplasm for utilization in conservation biotechnologies [17,18]. A particular approach to ex situ conservation is through biobanks, which are repositories of cryopreserved genetic resources [19,20,21].

The imminent worldwide mass extinction requires immediate conservation strategies, with a particular emphasis on the preservation of genetic resources, even for currently non-threatened species. Furthermore, there is a need to develop innovative and increasingly efficient methodologies for the collection, preservation and utilization of these resources. In a pioneering study, Katayama et al. [17] successfully reprogrammed cells from endangered Japanese birds, collected eight to twelve years prior. Building on this, our research presents a method for isolating stem cells from developing feathers and characterizing them.

## 2. Experimental Design

The protocol was established after the collection and culture of follicular cells from 18 wild birds (5 psittaciformes, 4 galliformes, 2 struthioniformes, 1 passerine, 1 strigiformes, 1 cariamiformes, 1 cuculiformes, 1 caprimulgiformes, 1 accipitriformes and 1 cathartiformes), carried out with success, by plucking the feathers of birds that died, and isolating the follicular cells. This method represents a more practical approach to the acquisition of genetic material from wild birds than biopsy or the collection of embryos and gametes. The birds came from our partners: the Veterinary Hospital of the São Paulo University, Pirassununga, Brazil; Zoo das Aves, Poços de Caldas, MG, Brazil; and Clínica Arca de Noé, Pirassununga, SP, Brazil (Table 1).

**Figure 1:**
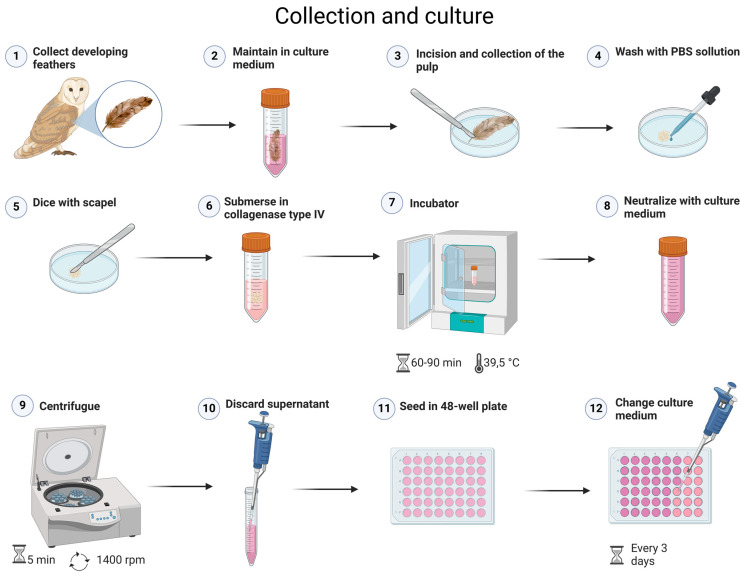
Experimental design of step-by-step feather pulp extraction procedures.

The feathers were stored in a refrigerated culture medium until the processing stage. The feather calamus was opened and its pulp extracted using a stereomicroscopic LABOMED Luxeo 4D model in a Petri dish. The tissue was washed on three occasions in phosphate-buffered saline (PBS), 2% penicillin/streptomycin (Sigma-Aldrich, New York, NY, USA) and 1% amphotericin B, and then finely diced. The enzymatic digestion of the tissue was initiated by submerging it in collagenase type IV, which was incubated at 39.5 °C for 60–90 min. Subsequently, neutralization was conducted with a culture medium, followed by centrifugation at 1400 rpm for five minutes, after which the supernatant was discarded. Subsequently, the resulting solution was then seeded in a 48-well culture plate. The culture medium was composed of Dulbecco’s Modified Eagle’s Medium, 15% fetal bovine serum, 1% penicillin/streptomycin and 0.5% amphotericin B, and was changed every three days (Figure 1). It was possible to collect viable cells from feathers refrigerated in the medium for 24 h. These data opened up possibilities for collections hours after the bird’s death.

Passages were performed with 5% EDTA for 5 min in an incubator at 39.5 °C, followed by neutralization with culture medium and centrifugation to remove the supernatant, thus enabling the seeding in a new plate.

### 2.1. Materials

1. Adson serrated tip tweezers
2. Rubbing ethyl alcohol, 70%
3. Bonn miniature iris scissors or Noyes c (ABC^®^, Sydney, Australia; Cat. No. RH636004)
4. Scalpel (Sigma-Aldrich^®^; Cat. No. S2896)
5. Conical centrifuge tube, 15 mL (Perfect, Vancouver, WA, USA; Cat. No. 216090015)
6. Pipette and pipette tips (Olen-Kasvi^®^, Olen, Belgium; Cat. No. K31-10000)
7. Pasteur pipette (Olen-Kasvi^®^; Cat. No. K30-300S)
8. Cell culture dish, polystyrene treated, 35 mm × 10 mm (Corning^®^, Corning, NY, USA; Cat. No. CLS430165)
9. Dulbecco’s Modified Eagle’s Medium (DMEM) (Gibco^®^, Grand Island, NY, USA; Cat. No. 12100046)
10. Phosphate-buffered saline (PBS, Boston, MA, USA)
11. Penicillin/streptomycin (Gibco^®^; Cat. No. 15140122)
12. Amphotericin B (Gibco^®^; Cat. No. 15290026)
13. Fetal bovine serum (Gibco^®^; Cat. No. 12657029)
14. Collagenase type IV (Sigma-Aldrich^®^; Cat. No. C4221G)
15. Cell culture plates, 6- and 12-well (Corning^®^; Cat. No. CLS3548)

### 2.2. Equipment

16. Luxeo 4D stereomicroscope (LABOMED^®^, Los Angeles, CA, USA; Cat. No. 4145000)
17. Biological culture hood (BIOSEG 12^®^, Cairo, Egypt; Cat. No. 1EAC3772)
18. Centrifuge for falcon tubes (Kindly^®^ KC4 model, Kent, UK; Cat. No. KC 4-1)
19. Cell culture incubator (Thermo Scientific^®^—3 Water Jacketed CO_2_ Incubator, Waltham, MA, USA; Cat. No. 4110)
20. Inverted TCM400 microscope (LABOMED^®^ TCM400; Cat. No. 7125000)
21. EVOS M5000 microscope (Thermo Scientific^®^; Cat. No. AMF5000SV)

## 3. Procedure

### 3.1. Tissue Collection

#### 3.1.1. Preparation of the Collection Materials

1. To guarantee sterile conditions and avoid contamination, clean and disinfect the laminar flow and stereomicroscope hood with 70% ethanol.
2. Disinfect and autoclave tools (e.g., scissors and tweezers) before use.
3. Prepare the culture medium in advance under a laminar flow hood, under sterile conditions: Dulbecco’s Modified Eagle’s Medium with 15% fetal bovine serum, 1% penicillin/streptomycin and 0.5% amphotericin B. Keep refrigerated and warm to 37 °C before use.
4. Prepare the washing solution in advance under a laminar flow hood, under sterile conditions: phosphate-buffered saline with 2% penicillin/streptomycin and 1% amphotericin B.
5. Prepare the collagenase type IV in a laminar flow hood under sterile conditions and protected from light: dissolve collagenase type IV in Dulbecco’s Modified Eagle’s Medium at a concentration of 1.5 mg/mL and filter-sterilize using a 0.22 µm pore size filter unit.
6. Prepare the conical centrifuge tube with a culture medium.

#### 3.1.2. Extraction of Developing Feathers

**Figure 2:**
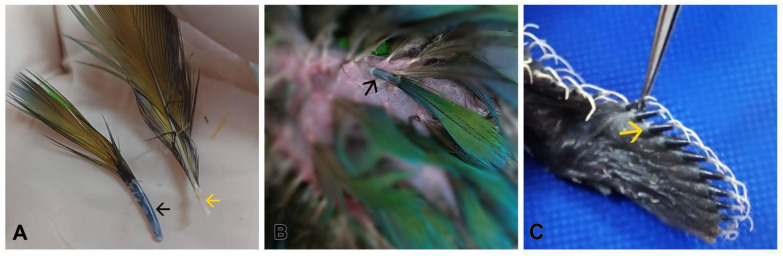
Photographs highlighting the stage of the feathers collected. (**A**) Feathers of *Ara ararauna*, adequate for use with pulp (black arrow) and inadequate without pulp (yellow arrow), and (**B**) developing feather containing sheath and pulp. (**C**) feathers on the wing of a chick of the species *Guira guira* (yellow arrow).

1. Select developing feathers which present the feather sheath surrounding them (Figure 2).

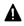

**Figure 3:**
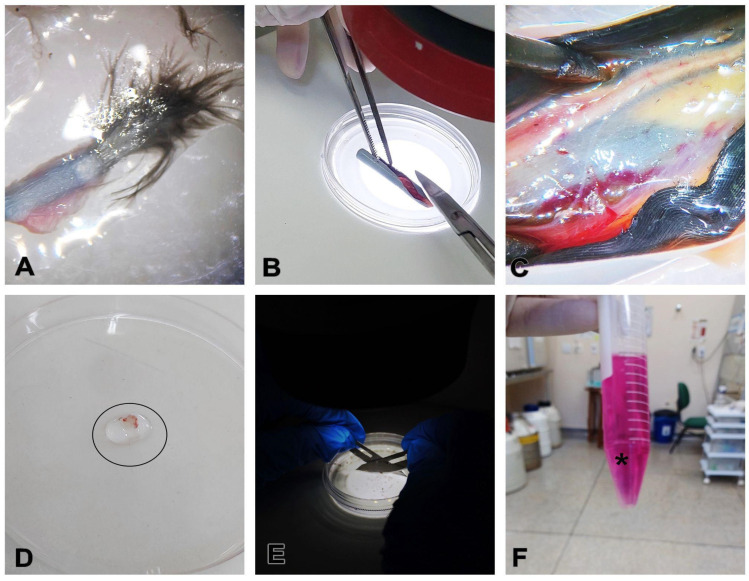
Feather pulp extraction protocol. (**A**) In a biosafety cabinet, place the feathers already sectioned in the calamus region in a sterile 60 mm polystyrene Petri dish. (**B**) Using a stereomicroscope for better visualization, with the aid of small sterile surgical scissors and tweezers, make a longitudinal incision in the calamus of the feather to expose the pulp, (**C**) scrape the pulp from the calamus and place it in another Petri dish containing PBS. (**D**) Wash the sample 3 times, for 2 min each time, with the washing solution (circle), (**E**) mechanically section using scalpel blades, and then (**F**) transfer the contents to a falcon tube and immerse in collagenase IV for enzymatic digestion at a ratio of 1:1, at a concentration of 1.5 mg/mL (asterisk).

CRITICAL STEP: Mature feathers present hollow calamus and are not a source of stem cells due to the regression of the feather follicle during the feather development cycle. Make sure to collect developing, immature feathers. The difference between developing and mature feathers is shown in Figure 3A.

OPTIONAL STEP: Wet the feather area to facilitate the extraction of the feather.

2. Remove by plucking the developing feathers.

OPTIONAL STEP: A small incision around the feather may help in the extraction of the feather.

3. Store plucked feathers at 4 °C in the conical centrifuge tube with medium until use.

#### 3.1.3. Extraction of the Feather Pulp

1. In a biological safety cabinet, place the feathers already sectioned in the calamus region in a sterile 60 mm polystyrene Petri dish (Figure 3A).
2. Using a stereomicroscope for better visualization, with the aid of small sterile surgical scissors and tweezers, make a longitudinal incision in the calamus of the feather to expose the pulp (Figure 3B,C).
3. Scrape the pulp off the calamus and transfer to another Petri dish with PBS (Figure 3C,D).
4. Wash the sample 3 times, for 2 min each time, with the washing solution.
5. Mechanically dice the content as finely as possible using scalpel blades, then transfer the contents to a falcon or Eppendorf tube and immerse in collagenase IV for enzymatic digestion at a ratio of 1:1 at a concentration of 1.5 mg/mL (Figure 3E,F).

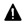

CRITICAL STEP: Before cutting, remove any blood or fragments from the content.

6. Leave in the incubator at 39.5 °C from 60 to 90 min. Shake vigorously every 15 min to break the remaining tissue fragments.
7. Having most of the solid fragments digested, neutralize the collagenase with the same amount of culture medium.
8. Centrifuge the tubes at room temperature at 1400 rpm for 5 min.
9. Remove the supernatant and dispose of it.
10. Add 2 mL of culture medium to the remaining cells in the conical centrifuge tube.
11. Sow 500 µL of the extracted feather cells in a 6/12-well plate and incubate it in an incubator at 39 °C with 5% CO_2_.

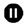

PAUSE STEP

12. Change the medium at 72 h intervals.

### 3.2. Follicle Cell Culture

#### 3.2.1. Changing the Culture Medium

The following procedure is to be carried out at 72 h intervals throughout the duration of the FFC.

1. Clean and sterilize the laminar flow hood, microscope and tools.
2. Prepare the culture medium.
3. Remove the plate to be changed from the incubator and observe the follicle cells under the microscope.

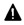

CRITICAL STEP: Before taking the culture plate into the laminar flow hood, it is important to observe the adhesion of the cells to the plate under the microscope. This can be easily visualized by causing a slight trembling of the desk where the microscope is located. If the cells are fully fixed, there will be no movement; in this case, all of the culture medium will be changed. If some cells respond to the movement, only 50% of the medium will be removed from the well to avoid discarding viable cells.

4. Place the plate in the laminar flow hood and carefully remove the culture medium with a pipette.
5. Replace the culture medium with the same quantity that was removed from each well.
6. Return the plate to the incubator.

#### 3.2.2. Passage

In order to ensure the efficacy of this procedure, it is imperative to monitor the extent of cell confluence, which must not exceed 80%.

1. Prepare the solution of EDTA 5 M in a 1:1000 dilution in PBS.
2. Place the plate in the laminar flow hood.
3. Remove the culture medium with a pipette.
4. Add 1–2 mL of the EDTA solution to the wells and return the plate to the incubator for 5 min.
5. After 5 min, observe the cell adherence. This can be easily visualized by causing a slight trembling of the desk where the microscope is located.

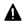

CRITICAL STEP: To continue the passage protocol, the cells must no longer be attached to the bottom of the well, meaning that they can be collected and transferred to a conical centrifuge tube for the next step. If the cells are not fully detached, it is recommended to place them back in the incubator for 2 min and gently pipette the medium against the well’s wall.

6. If all the cells are detached from the bottom of the well, transfer the contents to a 15 mL conical centrifuge tube.
7. Neutralize with the same amount of culture medium.
8. Centrifuge the conical centrifuge tube at 1400 rpm for 5 min.
9. Remove the culture medium from the wells and transfer approximately 2 mL to a conical centrifuge tube.
10. Sow the cells in a 48-well plate and complete each well with 100–200 µL of culture medium. Incubate the plate at 39.5 °C with 5% CO₂.

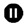

PAUSE STEP

11. Change the medium at 72 h intervals.

## 4. Expected Results

**Figure 4:**
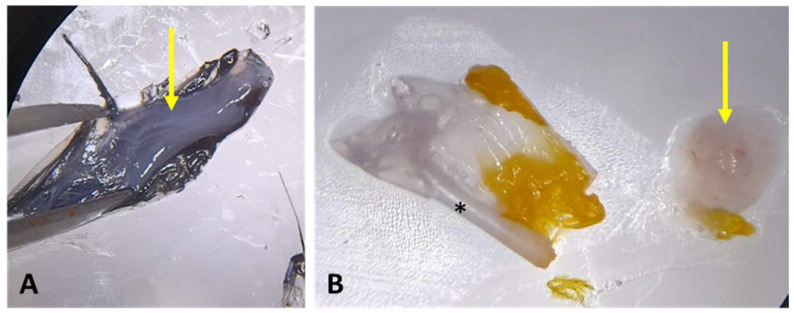
The images illustrate the collection of feather pulp. (**A**) The feather pulp of an *Aburria cujubi* is visible (yellow arrow). (**B**) The sectioned calamus (asterisk) and feather pulp (yellow arrow) of an *Ara ararauna* are shown.

The results show that the feather pulp is gelatinous and exhibits no discernible variation according to the order of the wild birds. However, the color can vary between black, gray, ice and yellow (Figure 4). The feather fragments continue to dissipate progenitor cells at varying times, ranging from 24 to 72 h.

The success of the culture may be influenced by the time elapsed between the animal’s death and processing, as well as the storage method employed. Optimal results are obtained when the feathers are collected up to six hours after death and stored in a refrigerated culture medium for the shortest possible time until processing (Table 2).

**Figure 5:**
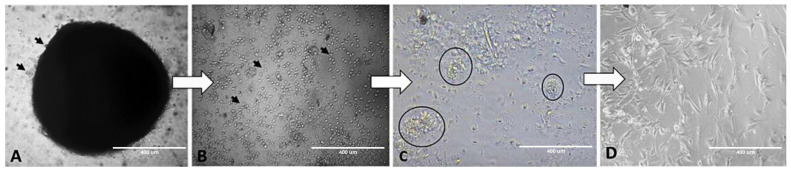
Photomicrographs showing the progression of feather follicle cell culture in wild birds. Immediately upon placement of the explant on the well plate, the cells begin to grow (**A**, arrows). After 3 days, more cells were growing and were observed to acquire an oval shape (**B**, arrows). They then began a process of cell clustering (**C**, circles), to conclude the process by transforming into fibroblastoid-shaped cells (**D**). The images show cells of the following species: (**A**) *Aburria cujubi*, (**B**) *Cariama cristata*, (**C**) *Dryocopus lineatus* and (**D**) *Ara ararauna*. Scale bar = 400 µm.

The cellular growth of feather FFC explants was observed as early as 24 h after their isolation. Cell cultures presented homogenous, rounded cells devoid of any discernible nucleus. After 48 to 240 h (depending on species) the cells showed the formation of clusters followed by some stellate cells with fibroblast-like characteristics. In this method, the cell cultures reached 70–80% confluency in 5 to 14 days (depending on species) of incubation. At this point, the cultures could be split into well plates or chamber slides according to the planned experiment (Figure 5).

**Figure 6:**
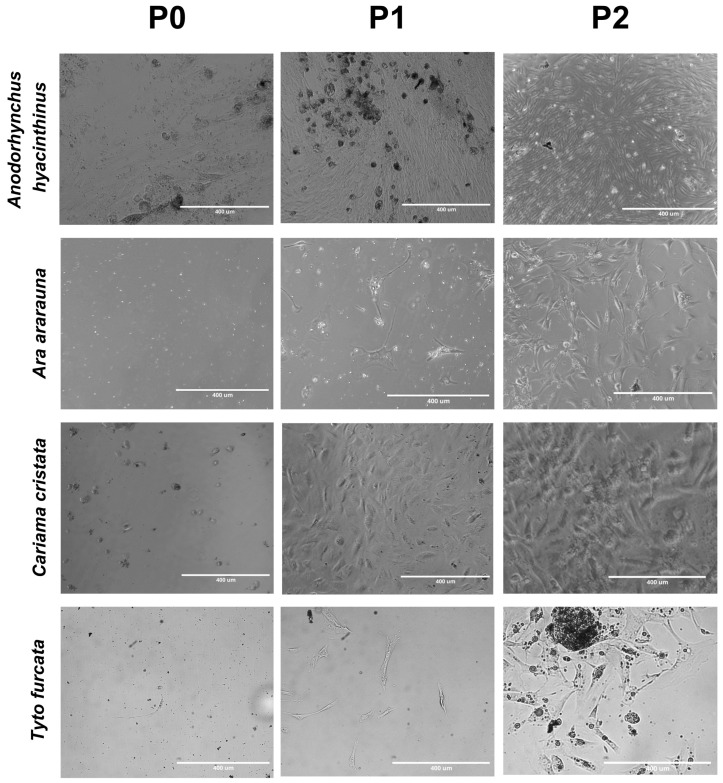
Photomicrography of wild bird FFSC morphology. The images show cells from *Anodorhynchus hyacinthinus*, *Ara ararauna*, *Cariama cristata* and *Tyto furcata* at different passages (P0, P1 and P2). These cells adhered to the surface of the plate within two days of isolation, reaching 70–80% confluence in 2 to 3 weeks. Morphological differences and particularities in culture maintenance were observed between the species, suggesting intrinsic variability. Scale bar = 400 µm.

After the isolation of feather follicular cells with collagenase, we observed cell attachment to the surface of the 12- or 6-well plate from two days of isolation onwards. The cells reached 70–80% confluency in 2 to 3 weeks of incubation. At this point, the cultures could be split to well plates or chamber slides according to the envisaged experiment (Figure 6).

The follicle cells in culture demonstrated varying maintenance times with the preservation of morphological quality, ranging from 96 to 336 h. In the initial passage (P0), the cells show a more heterogeneous morphology, which evolves into more uniform characteristics in subsequent passages, with an evident capacity for cell adhesion and proliferation. This variability may be related to factors intrinsic to the species, as observed in *Ara ararauna*, whose sample took around seven hours to process before being cultured. Despite the delay in manipulation, the cells maintained stable morphological characteristics, suggesting an adaptation specific to this species (Figure 6).

The difficulty of maintaining the cultivation of follicular cells from wild birds is linked to the difficulty in obtaining biological materials and is intrinsically linked to the quantity of tissue obtained. This factor is related to the size of the animal and the availability of developing feathers due to molting or regeneration after plucking, as matured feathers are no longer a source of these cells due to the regression of follicular cells into the dermal papilla during the natural feather follicle regeneration cycle.

## 5. Conclusions

This article presents a detailed protocol for the isolation and maintenance of follicular cells from 10 different species of wild birds and highlights the stem potential of the cells through the morphology presented. It demonstrates that follicular cells can be obtained from birds that die, without the necessity of euthanasia for the collection of material. Our work is aligned with the Sustainable Development Goal (SDG) 15, “Life on Land”. This study provides novel insights into the conservation of genetic material from wild birds, offering a comprehensive approach to the development of techniques for cell extraction, maintenance, proliferation and cryopreservation. This approach underscores the potential for a novel source of genetic material preservation, with the possibility of serving as a valuable tool for the conservation of endangered bird species. The formation of a genetic resource bank for the maintenance of genetic material, analyses, and studies of molecular characterizations is a key recommendation from this study.

## Figures and Tables

**Table 1:** Species used to establish the follicle cell culture protocol.

Order	Family	Scientific Name	Representative Picture	Conservation Status	Collection Date
Accipitriformes	Accipitridae	* Geranospiza caerulescens *	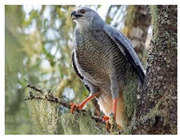 https://ebird.org/species/crahaw (accessed on 28 November 2024).	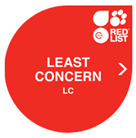 https://www.iucnredlist.org/species/22695729/168785689 (accessed on 28 November 2024)	21 November 2024
Apodiformes	Trochilidae	* Eupetomena macroura *	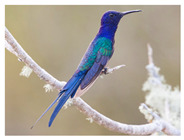 https://ebird.org/species/swthum1 (accessed on 28 November 2024)	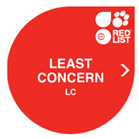 https://www.iucnredlist.org/species/22687094/263628596 (accessed on 28 November 2024)	6 April 2022
Caprimulgiformes	Caprimulgidae	*Nyctidromus albicollis*	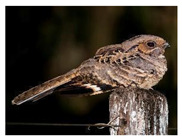 https://ebird.org/species/compau (accessed on 28 November 2024)	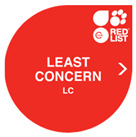 https://www.iucnredlist.org/species/22689731/168860360 (accessed on 28 November 2024)	1 November 2024
Cariamiformes	Cariamidae	*Cariama cristata*	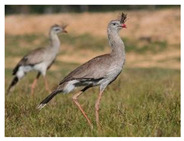 https://ebird.org/species/relser1 (accessed on 28 November 2024)	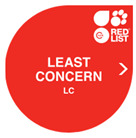 https://www.iucnredlist.org/species/22692205/263629033 (accessed on 28 November 2024)	20 March 2023
Cathartiformes	Cathartidae	* Coragyps atratus *	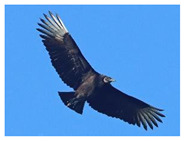 https://ebird.org/species/blkvul (accessed on 28 November 2024)	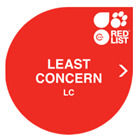 https://www.iucnredlist.org/species/22697624/93624950 (accessed on 28 November 2024)	1 November 2022
Cuculiformes	Cuculidae	*Guira guira*	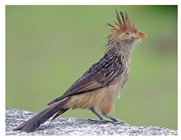 https://ebird.org/species/guicuc1 (accessed on 28 November 2024)	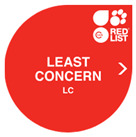 https://www.iucnredlist.org/species/22684441/263682441 (accessed on 28 November 2024)	1 November 2024
Galliformes	Cracidae	*Pauxi mitu*	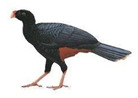 https://ebird.org/species/alacur1 (accessed on 28 November 2024)	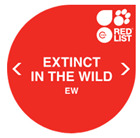 https://www.iucnredlist.org/species/22678486/132315266 (accessed on 28 November 2024)	3 May 2024
		* Pipile cujubi *	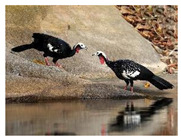 https://ebird.org/species/rtpgua1 (accessed on 28 November 2024)	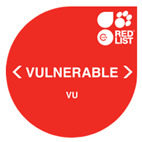 https://www.iucnredlist.org/species/22678422/194242016 (accessed on 28 November 2024)	8 April 2022
	Phasianidae	*Gallus gallus*	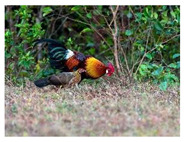 https://ebird.org/species/redjun (accessed on 28 November 2024)	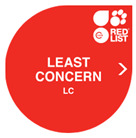 https://www.iucnredlist.org/species/22679199/263732457 (accessed on 28 November 2024)	6 July 2022
		*Lophura edwardsi*	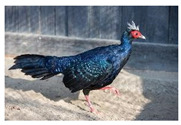 https://commons.wikimedia.org/wiki/File:Ba%C5%BEant_Edwards%C5%AFv.jpg (accessed on 29 November 2024)	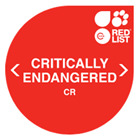 https://www.iucnredlist.org/species/45354985/249120158 (accessed on 29 November 2024)	8 April 2022
		*Meleagris gallopavo*	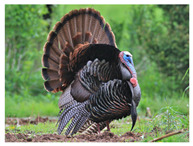 https://ebird.org/species/wiltur (accessed on 29 November 2024)	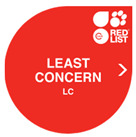 https://www.iucnredlist.org/species/22679525/132051953 (accessed on 29 November 2024)	22 August 2023
Passeriformes	Icteridae	*Cacicus cela*	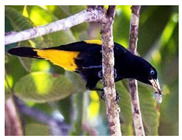 https://ebird.org/species/yercac1 (accessed on 29 November 2024)	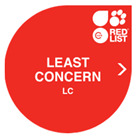 https://www.iucnredlist.org/species/103792683/138350097 (accessed on 29 November 2024)	21 April 2022
Psittaciformes	Psittacidae	*Amazona festiva*	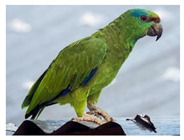 https://ebird.org/species/fespar1 (accessed on 29 November 2024)	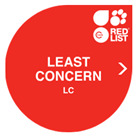 https://www.iucnredlist.org/species/22727626/209890756 (accessed on 29 November 2024)	19 July 2022
		*Anodorhynchus hyacinthinus*	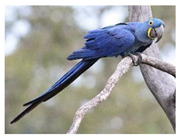 https://ebird.org/species/hyamac1 (accessed on 29 November 2024)	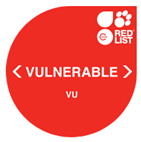 https://www.iucnredlist.org/species/22685516/93077457 (accessed on 29 November 2024)	3 May 2024
		*Ara Ararauna*	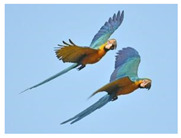 https://ebird.org/species/baymac (accessed on 29 November 2024)	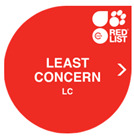 https://www.iucnredlist.org/species/22685539/131917270 (accessed on 29 November 2024)	5 August 2022
		*Ara rubrogenys*	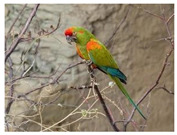 https://ebird.org/species/refmac1 (accessed on 29 November 2024)	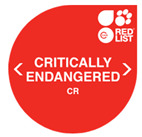 https://www.iucnredlist.org/species/22685572/196567391 (accessed on 29 November 2024)	18 April 2022
		*Pyrrhura frontalis*	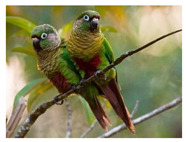 https://ebird.org/species/mabpar (accessed on 29 November 2024)	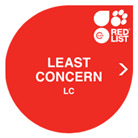 https://www.iucnredlist.org/species/22685793/93088076 (accessed on 29 November 2024)	8 April 2022
Strigiformes	Tytonidae	*Tyto furcata* (old *Tyto alba*)	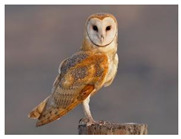 https://ebird.org/species/brnowl (accessed on 29 November 2024)	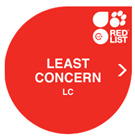 https://www.iucnredlist.org/species/22688504/155542941 (accessed on 29 November 2024)	31 August 2022
Struthioniformes	Tinamidae	*Crypturellus noctivagus*	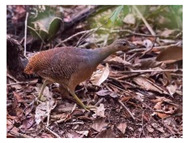 https://ebird.org/species/yeltin1 (accessed on 29 November 2024)	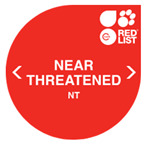 https://www.iucnredlist.org/species/22678217/221182790 (accessed on 29 November 2024)	21 April 2022
		*Crypturellus obsoletus*	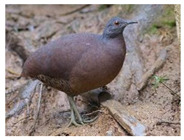 https://ebird.org/species/brotin1 (accessed on 29 November 2024)	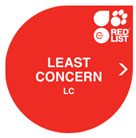 https://www.iucnredlist.org/species/22678176/234006619 (accessed on 29 November 2024)	16 April 2022

**Table 2:** Comparative characteristics of wild birds’ cells in culture.

Species	Collection Timepoint	Scattering from the Fragment	Cell Aggregate	Scattered Cells	Fibroblast-like Morphology (days)	Presence of Rounded Cells with No Apparent Nucleus	Adhesion to Plate (days)
* Pipile cujubi *	After euthanasia	+	+	+	+	+	14
*Lophura edwards*	After euthanasia	+	+	+	−	+	4
*Gallus gallus*	8 h after death	−	−	−	−	−	absent
* Amazona aestiva *	5 h after death	+	+	+	−	+	absent
* Ara arauna *	2 h after death	+	+	+	5	+	5
* Eupetomena macroura *	24 h after death	+	−	+	absent	+	3
* Tyto furcata *	6 h after death	+	+	+	22	+	16
* Cariama cristata *	6 h after death	+	+	+	3	+	3
* Meleagris gallopavo *	24 h after death	+	+	+	3	+	3

Legend: A comparative description of the characteristics of wild bird cells in culture is provided. The presence of a given characteristic is indicated by (+), while its absence is indicated by (−).

## Data Availability

All data are provided within the main text. Further data inquiries are available from the authors upon reasonable request.
